# Adaptive urban traffic signal control based on enhanced deep reinforcement learning

**DOI:** 10.1038/s41598-024-64885-w

**Published:** 2024-06-19

**Authors:** Changjian Cai, Min Wei

**Affiliations:** School of Electronic Engineering, Xi’an Shiyou University, Xi’an, 710065 Shaanxi China

**Keywords:** Urban traffic, Signal light control, Deep reinforcement learning, Urban traffic, Computer science, Electrical and electronic engineering

## Abstract

One of the focal points in the field of intelligent transportation is the intelligent control of traffic signals (TS), aimed at enhancing the efficiency of urban road networks through specific algorithms. Deep Reinforcement Learning (DRL) algorithms have become mainstream, yet they suffer from inefficient training sample selection, leading to slow convergence. Additionally, enhancing model robustness is crucial for adapting to diverse traffic conditions. Hence, this paper proposes an enhanced method for traffic signal control (TSC) based on DRL. This approach utilizes dueling network and double q-learning to alleviate the overestimation issue of DRL. Additionally, it introduces a priority sampling mechanism to enhance the utilization efficiency of samples in memory. Moreover, noise parameters are integrated into the neural network model during training to bolster its robustness. By representing high-dimensional real-time traffic information as matrices, and employing a phase-cycled action space to guide the decision-making of intelligent agents. Additionally, utilizing a reward function that closely mirrors real-world scenarios to guide model training. Experimental results demonstrate faster convergence and optimal performance in metrics such as queue length and waiting time. Testing experiments further validate the method's robustness across different traffic flow scenarios.

## Introduction

With the flourishing development of the global automotive industry, the prevalence of private cars continues to rise, thus resulting in frequent traffic congestion^1^. According to statistics, in 2019, the total greenhouse gas emissions resulting from congestion reached 36 million tons^2^, with over half generated by light vehicles, causing adverse effects on the economic development of the United States during peak traffic congestion periods^3^. Traffic congestion can be addressed through improvements in public transportation systems, road expansions, and the implementation of traffic management policies^4^. However, factors such as geographical conditions, government policies, financial constraints, technological deficiencies, and societal acceptance limit the effectiveness of these methods in alleviating traffic congestion, thus necessitating active exploration of more efficient solutions.

Existing infrastructure cannot be changed, urban TSC is an economical and efficient way to address traffic congestion^5^. Today, urban TS are typically optimized using fixed-time control (FTC), induction control, and Adaptive Traffic Signal Control (ATSC) methods. FTC is a repetitive pattern that does not change with real-time traffic conditions. Its cycle continues regardless of dynamic traffic changes in the area^6^. Induction control methods operate TS based on data from loop detectors. However, during the process of collecting and analyzing traffic flow data, the actual conditions at intersections may have changed, induction control methods unable to fully meet dynamic traffic demands^7^. In contrast, ATSC dynamically adjusts signal timing strategies based on real-time traffic information to reduce potential congestion in saturated road networks^8^. Early ATSC methods addressed optimization issues by seeking effective coordinated control strategies, such as SCOOT^9^ and TUC^10^.

Reinforcement Learning (RL) is a method that holds promise for adaptively adjusting TSC strategies based on real-time traffic conditions^11^. Deep Learning is a machine learning method that learns data features through hierarchical abstraction^12^. The combination of deep learning and RL is termed DRL^13^. The development of DRL provides additional technical support for ATSC, further driving the success of intelligent transportation^2,14^. Despite some successes in the field of TSC, DRL also faces notable drawbacks and challenges. For instance, DRL suffers from inefficient training sample selection because uniform sampling is unscientific due to the varying importance of each sample^15^, leading to slow convergence. Additionally, enhancing model robustness is crucial for adapting to diverse traffic conditions, as real-world traffic flows are dynamically changing^16^. Therefore, it is necessary to further enhance training speed and improve model robustness. This paper makes the following contributions:

- A TSC model is proposed in this paper, which integrates a comprehensive strategy involving dueling network, noisy network, prioritized experience replay, and double q-learning, and is referred to as PN_D3QN.
- A safer phase-cycle action space is designed for intelligent agent action decision-making, with a more realistic reward function guiding model training. This approach considers vehicle positions, velocities, and the current phase state space to extract traffic environment features.
- Multiple traffic flow scenarios are used to validate the effectiveness and robustness of the proposed model.

## Related work

Many methods have been proposed for constructing TSC strategies. For example, TS controllers based on fixed-time cycles^17^select the next phase to display in a sequence of cycles, with each phase having a fixed duration. This simple cycle-based TSC serves as a baseline and is the most widely used method currently. Researchers have also developed the Max Pressure methods^18^, which controls TS by calculating the number of vehicles upstream and downstream of intersections, performing well in traditional models. Additionally, some traditional optimization models, such as programming^19^ and fuzzy logic methods^20^, have been used for intersection signal control.

In recent years, artificial intelligence has seen significant advancements, and RL algorithms have emerged as a key direction for future TSC development^14,19^. These algorithms can learn and adjust control strategies based on environmental feedback, displaying strong adaptability. Early RL-based signal control methods used Q-tables^21–24^. However, Q-tables are limited to solving dynamic programming problems with finite state dimensions. Facing complex TSC problems, deep neural networks are often used due to their powerful state representation and mapping capabilities. Among them, the deep neural network used to estimate the Q-value of discrete actions is called Deep Q Network (DQN)^25^. Research on TSC based on DQN has attracted considerable attention^26–29^. DQN is a value iteration-based model. Additionally, some researchers use policy iteration to control traffic signals^30,31^.

When applying DRL methods to TSC, it's necessary to define states, actions, and rewards. States generally refer to the distribution of vehicles at a specific intersection at a certain moment, such as queue length, vehicle density, vehicle count, and matrix representation of images^15^. Actions usually involve phase selection or dynamically adjusting the green light time within a phase, and their design is related to signal phase. The ultimate goal of TSC is to reduce traffic congestion and improve operational efficiency. Therefore, researchers can use indicators evaluating intersection performance or linear combinations of several indicators as rewards, such as queue length, waiting time, delay^32^. However, in the real world, some indicators are inconvenient to measure in real-time, such as vehicle waiting time, delay, etc. In our approach, states are defined as matrices of vehicle positions and speeds, along with signal phase; actions are defined as green light time within the phase; reward is defined as queue length. After defining these concepts, it's necessary to select an algorithm for training deep neural networks. The most commonly used algorithm currently is DQN and its variants^26,28,33–36^. To enhance the performance of the DQN algorithm, we have also adopted a series of state-of-the-art techniques, including dueling network^37^, double q-learning^38^, prioritized experience replay(PER)^39^, and noise injection^40^.

## Preliminary

### Environment

**Figure 1:**
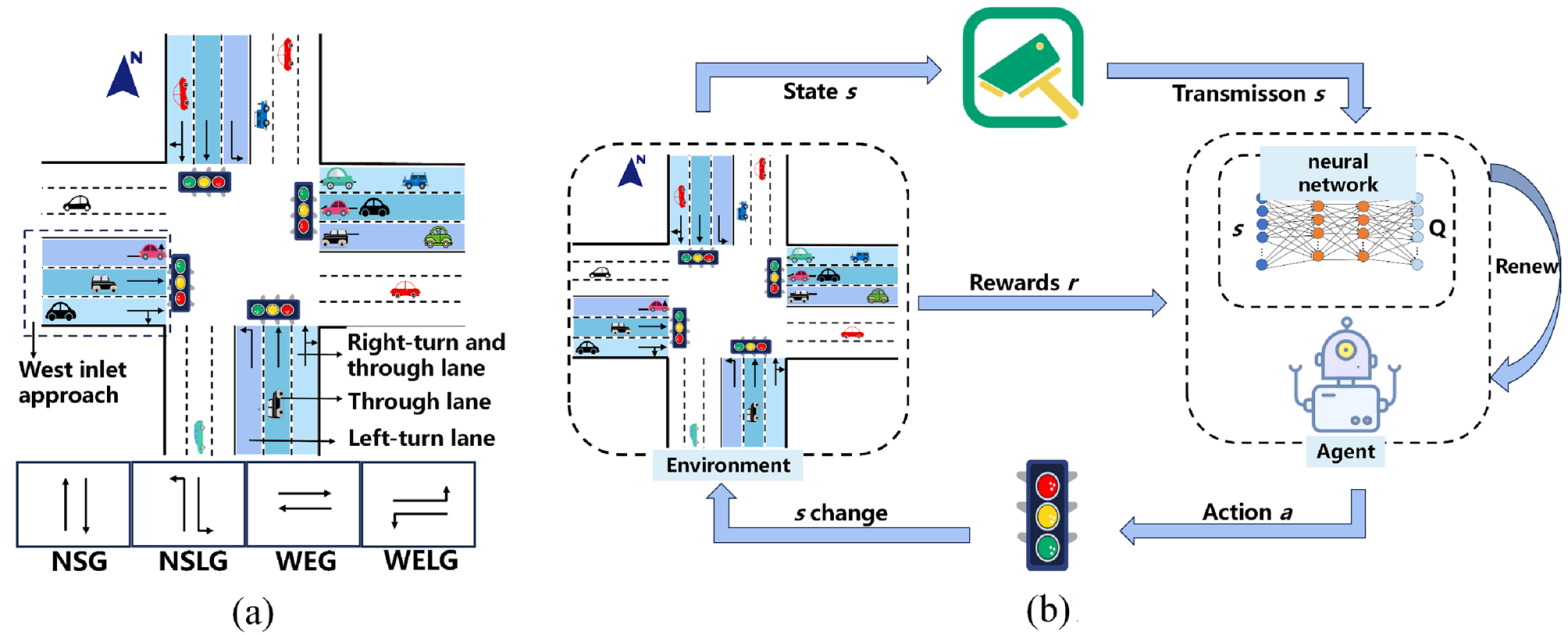
Urban intersections and TSC model. (**a**) Urban intersections. (**b**) TSC model.

Traffic congestion commonly occurs at urban intersections, as illustrated in Fig. 1a, thus this study focuses on the commonly encountered four-leg intersections in reality. Specifically, the research delves into TSC in single-intersection scenarios, using a standard four-leg intersection as an illustrative example. It is worth noting that the concepts and methods proposed are equally applicable to scenarios such as three-way or five-way intersections. There are the following definition.

- Inlet Approach: Vehicles enter the intersection area. There are four inlet approach in total: east inlet approach, south inlet approach, west inlet approach, and north inlet approach. Each inlet approach has three lanes, a left-turn lane, a through-lane, and a left-turn and through lane.
- Signal Phases: This study adopts the four most common signal phases in real life, namely, North–South through green light, North–South left-turn green light, East–West through green light, and East–West left-turn green light.

### Problem description

The TSC problem based on DRL can be modeled as a five-tuple <*S*, *A*, *P*, *R*, *γ*> of a Markov Decision Process^41^, referred to as an agent. The meanings are as follows:

- State Space *S*: The set of all states in the intersection environment, including information such as vehicle positions, speeds, and signal phases. Lowercase letter *s* represents the intersection state at a specific moment, where *s* ∈ *S*.
- Action Space *A*: The set of different control actions for the TS. Lowercase letter *a* represents a specific action, where *a* ∈ *A*.
- Transition Matrix *P*(*s*_t+1_*|s*_t_*,a*_t_): The collection of probabilities of the intersection transitioning from state *s*_t_ at time *t* to state *s*_t+1_ at time *t* + 1.
- Reward Set *R*: The set of rewards obtained for executing different actions *a* in different intersection states *s*. The reward *r* is computed using the designated reward function.
- Discount Factor *γ*: A hyperparameter controlling the importance of immediate rewards compared to future rewards. *γ* ∈ [0,1).

The TSC model based on DRL is depicted in Fig. 1b. Intersection cameras capture the traffic state *s*, which is then input to the neural network module of the agent. The neural network ultimately outputs Q-values, and the agent selects an action *a* based on the maximum Q-value to adjust the TS. During this process, the intersection state *s* changes according to the transition matrix *P*, and the environment returns a numerical reward *r* to the agent, marking the completion of a decision. The goal of the agent is to learn a policy *π* through repeated training and parameter updates, which maximizes the expected reward. This policy enables the agent to adaptively select the optimal action based on real-time intersection states, effectively addressing traffic congestion issues.

The learning strategy* π* can utilize the action-value function which represents the cumulative reward in the future after taking action *a* in state *s*. A higher cumulative reward implies a better strategy *π*. The specific definition is as follows:

1 $$Q_{\pi } (s_{t} ,a_{t} ) = E\left[ {{\text{r}}_{t} + \gamma r_{t + 1} + \gamma^{2} r_{t + 2} + \cdots |s_{t} = s,a_{t} = a} \right] = E\left[ {\sum\limits_{k = 0}^{\infty } {\gamma^{k} r_{t + k} |} s_{t} = s,a_{t} = a} \right]$$

If a certain strategy consistently achieves the highest cumulative reward, then that strategy is considered the optimal strategy. Therefore, it is necessary to know the optimal action-value function. Its definition is as follows:

2 $$Q_{{}}^{ * } (s_{t} ,a_{t} ) = \arg \max Q_{\pi } (s_{t} ,a_{t} )$$

Once the agent learns the optimal action-value function, it means that it has mastered the optimal policy *π*.

Traditional RL uses Q-tables to approximate the optimal action-value function. However, this method has limitations when dealing with large state spaces, as allocating a table entry for each state-action pair is impractical. To overcome this challenge, DRL introduces neural networks as approximation functions, approximating the optimal action-value function through nonlinear mappings. The parameters of the neural network are iteratively updated through DRL algorithms, enabling it to flexibly handle complex environments and abstract states.

## TSC model

### State space

To fully utilize the powerful feature extraction and learning capabilities of convolutional networks, we employ discrete state encoding techniques^28,33,34,42^, defining states as the positions, velocities, and signal phases of vehicles. Therefore, the system has very high requirements for detectors. In addition to the two most common methods for acquiring traffic data, loop and video detectors, autonomous vehicles can also be utilized as “mobile detectors” to overcome these challenges. Define the state set *S*:

3 $$S = \left\{ {S_{1} ,S_{2} ,S_{3} } \right\}$$

**Figure 2:**
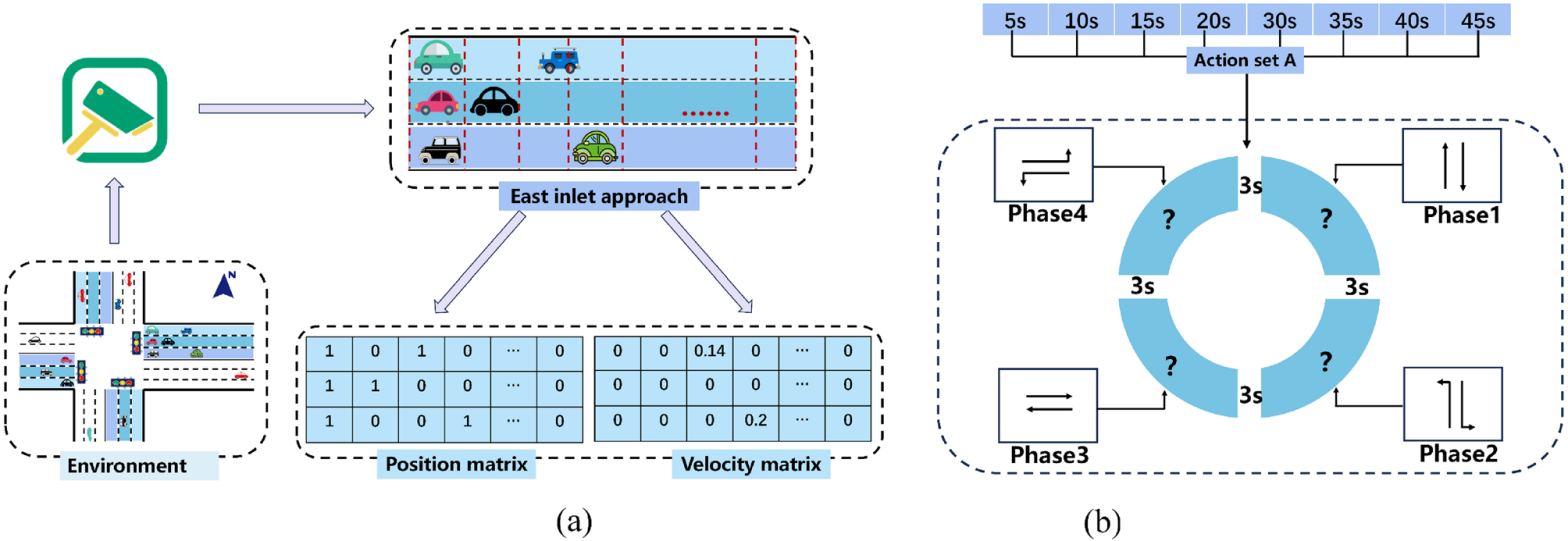
Example calculation of position-velocity matrix and signal light cycle period. (**a**) Example calculation of position-velocity matrix. (**b**) Signal light cycle period.

The example calculation of position-velocity matrix as shown in Fig. 2a, each lane is subdivided into multiple grids, with each grid capable of accommodating only one vehicle. In the position matrix, 1 indicates the presence of a vehicle at that grid position, while 0 indicates the absence of a vehicle. The number of digit 1 s within a grid reflects the number of vehicles stationary in that lane. Similarly, the numbers in the velocity matrix represent the speed information of vehicles. All lanes' traffic conditions can be represented by two matrices, one representing the position information of vehicles and the other representing the velocity information. Please note that we limit the detector’s coverage to approximately 147 m near the central area of the intersection. Considering vehicle lengths ranging from 4.5 to 5 m, with a minimum spacing of 2 m between vehicles, each grid spans 7 m. Consequently, the position state space size for the east inlet approach is 3 × 21, leading to a final position matrix state space size of 12 × 21. The velocity matrix size matches that of the position matrix.

4 $$S_{1} = \left[ {P_{n} ;P_{s} ;P_{w} ;P_{e} } \right]$$

5 $$S_{2} = \left[ {V_{n} ;V_{s} ;V_{w} ;V_{e} } \right]$$

where *P*_*n*_, *P*_*s*_, *P*_*w*_, *P*_*e*_, *V*_*n*_, *V*_*s*_, *V*_*w*_, and *V*_*e*_ represent the position matrices and velocity matrices of the lanes in the north, south, west, and east directions, respectively. The phase information of the traffic lights can be represented as:

6 $$S_{3} = (x_{1} ,x_{2} ,x_{3} ,x_{4} )^{T}$$

*x*_*1*_, * x*_*2*_, *x*_*3*_, *x*_*4*_ represent four phases, which are respectively for the north–south green light, north–south left turn green light, east–west green light, and east–west left turn green light. If *S*_*3*_ = (1,0,0,0), it represents that the current phase is the north–south green light, allowing vehicles in that direction to pass.

### Action set

A practically feasible action design should comprehensively consider the safety of all traffic participants. Although selecting the action design for the next phase at the intersection can significantly improve traffic efficiency, such a choice may modify the original phase sequence, thereby compromising the safety of drivers' travel. Therefore, the action space of this model is defined as the duration of green light for the current phase. The signal light cycle period as shown in Fig. 2b, (Phase 1, Phase 2, Phase 3, Phase 4, Phase 1, Phase 2…). At the beginning of each phase, the agent selects a duration of green light from the action set *A* = {5s,10s,15s,20s,30s,35s,40s,45s} based on the intersection state *s* to execute. After the green light of each phase ends, there will be a 3-s duration of yellow light to ensure traffic safety.

### Reward function

The reward function is used to evaluate the performance of the agent after executing an action. In TSC based on DRL, the design of the reward function should guide the agent to achieve superior training results, especially in alleviating traffic congestion. Due to the difficulty of obtaining metrics such as waiting time, travel time, and delay in real-time from traffic detection devices, this paper uses the queue length as the calculation indicator for the reward function. The reward function *r*_*t*_ is defined as the difference in queue lengths of all lanes between adjacent action time steps:

7 $$r_{t} = - \left( {\sum\nolimits_{i = 1}^{12} {q_{t + 1}^{i} } - \sum\nolimits_{i = 1}^{12} {q_{t}^{i} } } \right)$$

where $q_{t}^{i}$ represents the queue length of the i-th lane at action time step *t*. When *r*_*t*_ > 0, it indicates that the traffic condition improves after the agent executes an action, while *r*_*t*_ < 0 indicates deterioration.

## Algorithm and model training

### Dueling network and double q-learning

**Figure 3:**
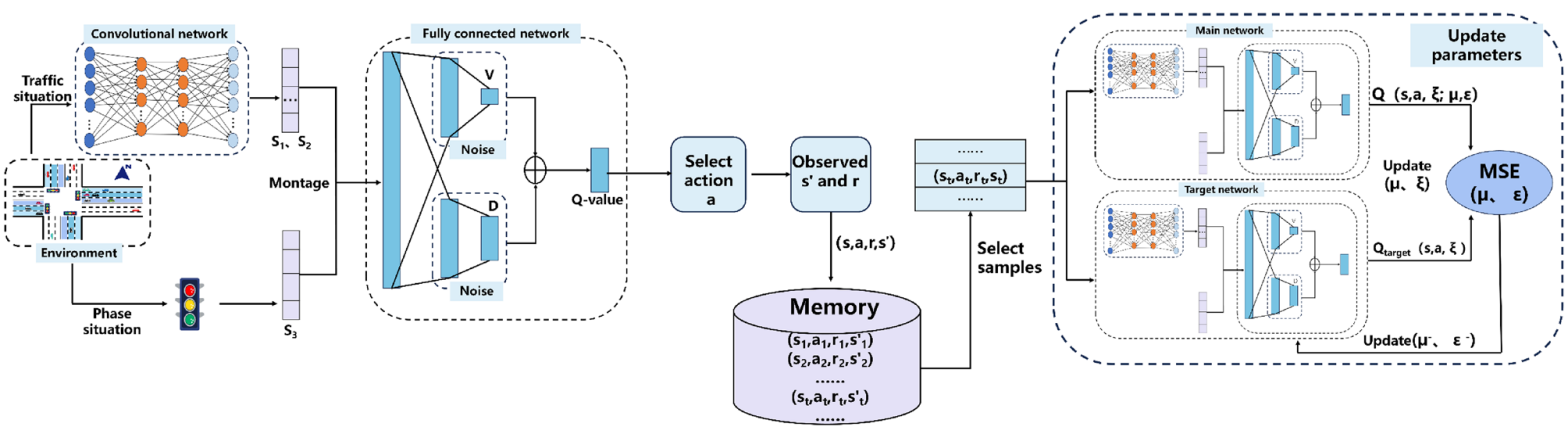
Model training process.

The dueling network^37^ and double q-learning^38^ algorithm is an optimized version of the DQN. It updates the neural network parameters to fit the function *Q*(*s*,*a*,*w*), thereby approximating the optimal action-value function Q*(*s*_*t*_,*a*_*t*_). Dueling network improves the neural network structure as shown in Fig. 3, one fully connected layer in the fully connected network is split into two parts, decomposing the predicted optimal action-value function *Q*(*s*,*a*,*w*) into the state value function *V*(*s*;*w*) and the advantage function *D*(*s*,*a*;*w*), to more accurately estimate the value of each action. At this point, the Q-value is calculated by the following equation:

8 $$Q(s,a;w) = V(s;w) + D(s,a;w) - \frac{1}{|D|}\sum\limits_{a} {D(s,a;w)}$$

where w represents the parameters of the neural network.

To update the parameters of the neural network, a target Q function is defined to assist in guiding the update, representing the target Q value when taking action *a* in state *s*. At this point, the loss function can be defined as follows:

9 $$J(w) = \frac{1}{m}\sum\limits_{i}^{m} {\left[ {Q_{t\arg et} (s,a) - Q(s,a;{\text{w}})} \right]^{2} }$$

where *m* represents the number of samples extracted from the experience pool. The uniqueness of double q-learning lies in choosing a main network to determine the optimal action, while another target network is used to evaluate the target Q value of that action. Where the target network has the same structure as the main network, but with different parameters *w*. At this point, it is calculated by the following equation:

10 $$Q_{t\arg et} (s,a) = r + \gamma Q\left. {\left( {s^{\prime},\arg \max Q(s^{\prime},a^{\prime};w);w^{ - } } \right)} \right)$$

where *w*^*-*^ represents the parameters of the target network, *s'* and *a'* are the state and action at the next time step, respectively. The gradient descent algorithm can use the loss value to update the parameters of the main network:

11 $$w \leftarrow w - \alpha \cdot \nabla_{w} J(w)$$

And the parameters *w*^*-*^ of the target network are updated by weighted averaging:

12 $${\text{w}}_{new}^{ - } = \tau w_{new} + (1 - \tau )w_{now}^{ - }$$

*τ* ∈ [0,1] is a hyperparameter that needs to be manually adjusted.

### Prioritized experience replay

To address the slow convergence speed in model training, the PER mechanism is introduced during the model training update process^39^. In the DQN algorithm, a uniform sampling method is used, where each sample has an equal probability of being selected. However, the importance of each sample is evidently different. Most samples represent normal traffic conditions, while samples leading to traffic congestion are more deserving of attention. Therefore, in PER, each sample is assigned a weight, and non-uniform sampling is conducted based on these weights. When the predicted optimal action-value function *Q*(*s*,*a*,*w*) deviates significantly from Q*(*s*_*t*_,*a*_*t*_), samples with a large |*Q*(*s*,*a*,*w*)- Q*(*s*_*t*_,*a*_*t*_)| should be assigned higher weights. Considering that Q*(*s*_*t*_,*a*_*t*_) cannot be directly obtained in practice, the target Q function is used as a substitute. The sample weight δ is defined as follows:

13 $$\delta_{j} = \left| {Q(s,a;w)_{j} - Q_{t\arg et} (s,a)_{j} } \right|$$

If the samples are sorted in descending order based on their weights, then the probability of sample *j* being selected can be defined as:

14 $$P_{j} = \frac{1}{rank(j)}$$

where *rank(j)* is the index of sample* j*. A smaller index indicates a larger weight *δ*, implying a greater disparity between the prediction of this sample and the actual target. Therefore, it should be given higher priority during training, resulting in a higher probability of being selected.

### Introduction noise

To enhance the adaptability of the model to different traffic flow scenarios, consider introducing noise in the fully connected network. Specifically, replace the original neural network parameters *w* with *µ* + *ε *⊙ *ξ*, where *µ*, *ε*, and *ξ* have the same shape as *w*. The symbols *µ* and *ε* represent the mean and standard deviation, respectively, which are parameters of the neural network learned from the samples. *ξ* represents random noise, with each of its elements independently sampled from the standard normal distribution N(0,1). The symbol “⊙” denotes element-wise multiplication. At this point, the function *Q*(*s*,*a*,*w*) is updated to:

15 $$Q\left( {s,a;w} \right) = Q\left( {s,a,\xi ;\mu ,\varepsilon } \right)$$

Accordingly, the loss function is updated to:

16 $$J(\mu ,\varepsilon ) = \frac{1}{m}\sum\limits_{i}^{m} {\left[ {Q_{t\arg et} (s,a) - Q(s,a,\xi ;\mu ,\varepsilon )} \right]^{2} }$$

The target network function is updated to:

17 $$Q_{t\arg et} (s,a) = r + \gamma Q(s^{\prime},\arg \max Q(s^{\prime},a^{\prime};\mu ,\varepsilon );\mu^{ - } ,\varepsilon^{ - } ))$$

The gradient descent updates parameters *µ* and *ε* of the main network:

18 $$\mu \leftarrow \mu - \alpha \cdot \nabla_{\mu } J(\mu ,\varepsilon )\;\;\;\varepsilon \leftarrow \varepsilon - \alpha \cdot \nabla_{\varepsilon } J(\mu ,\varepsilon )$$

The parameters *µ*^*-*^ and *ε*^*-*^ of the target network are updated according to the following equation:

19 $$\mu_{new}^{ - } = \tau \mu_{new} + (1 - \tau )\mu_{now}^{ - } \;\;\;\varepsilon_{new}^{ - } = \tau \varepsilon_{new} + (1 - \tau )\varepsilon_{now}^{ - }$$

By using *µ* + *ε *⊙ *ξ* to replace the original parameter *w*, the robustness of the model can be significantly improved. During training, if *ε* and *ξ* are not introduced, the obtained parameters will be *µ*. When the parameters strictly equal *µ*, the model can make a more accurate estimate of the optimal action-value function. However, when *µ* is perturbed, the model output may exhibit significant bias. By forcing the neural network to minimize the loss function *J*(*µ*,*ε*) during training with parameters containing noise, the model's resistance to interference is enhanced. As long as the parameters are within the neighborhood of *µ*, the model can make a reasonably accurate estimate of the optimal action-value function.

### Model training

To find the policy π that maximizes the expected reward, it is necessary to train the agent to learn the optimal action-value function Q*(*s*_t_, *a*_t_). The complexity of this function requires fitting with neural networks to effectively capture the nonlinear mapping relationship between the state space and actions. The neural network structure is divided into two parts: a convolutional network and a fully connected network. The model training process is illustrated in Fig. 3. The convolutional network extracts vehicle state information from the traffic environment and flattens it into position information *S*_*1*_ and velocity information *S*_2_. These are concatenated with phase information *S*_3_ to form a feature vector, which is then input into the fully connected network. The fully connected network ultimately outputs Q values, and the agent selects the action with the maximum Q value to control the traffic signal. As the traffic environment changes, the current state *s* and action *a*, as well as the next state *s*′ and received reward *r*, are stored in the memory in the form of a tuple (*s*,*a*,*r*,*s*′). When the number of samples in the memory reaches a certain threshold, PER is used to select a batch of samples for training the neural network parameters. Through repeated training to update the main network parameters *µ* and *ε*, they gradually fit a function that closely approximates the optimal action-value function Q*(*s*_t_,*a*_t_).

## Experimental setup and evaluation analysis

### Experimental setup

The experiments are conducted based on the SUMO traffic simulation platform. SUMO provides the traci interface for controlling TS and retrieving traffic information. Detector usage enables output of information such as queue lengths of vehicles at intersections. For the object of study depicted in Fig. 1a, each lane is 500 m long and 3 m wide. The development environment is the PyTorch framework, and the model parameters are listed in Table 1.

**Table 1:** Model parameters.

Parameters	Value	Parameters	Value
Optimizer	Adam	Simulation Duration *t*	4500 s
Hyperparameters *τ*	0.01	Sampling Batch Size B	128
Learning rate *α*	0.001	Episode N	200
Discount *γ*	0.95	Memory capacity D	50,000

### Traffic flow dataset

In order to make the simulation more realistic, all vehicle generation follows a random generation process, with vehicles randomly appearing from the entrance lanes and traveling to the intersection area. The length of each vehicle is 5 m, with a maximum speed of 13.9 m per second, a maximum acceleration of 0.8 m per second squared, a deceleration of 4.5 m per second squared, and a minimum distance between two vehicles of 2 m. The SUMO default car-following model is used during vehicle movement to ensure safe driving. The traffic flow is shown in Table 2. Scenario 1 is used to train the model to learn the optimal policy π, while the remaining scenarios are used to test whether the trained model can adapt to different traffic flow environments.

**Table 2:** Traffic flow data.

Config	Derections	Arrival rate (veh/s)	Starts (s)	End (s)
1	NS	0.3	0	3600
	WE	0.35	0	3600
2	NS/WE	0.2	0	3600
3	NS/WE	0.4	0	3600
4	NS	0.4	0	3600
	WE	0.3	0	3600
5	NS/WE	0.4	0	720
	NS	0.2	721	1440
	WE	0.3	721	1440
	NS	0.3	1441	2160
	WE	0.2	1441	2160
	NS/WE	0.25	2160	3600

### Evaluation metrics and comparative algorithms

The experimental objective is to accelerate the training speed of the DRL algorithm and improve intersection throughput efficiency by controlling phase signal lights. Therefore, the following metrics are used to evaluate the PN_D3QN algorithm:

- Cumulative Reward: The cumulative reward value of the DRL algorithm in one episode, where a higher value indicates better algorithm performance.
- Average Waiting Time: The average waiting time of vehicles on each lane in one episode, calculated by summing the waiting times of all lanes and dividing by the total number of lanes, measured in seconds.
- Average Queue Length: The average queue length of vehicles on each lane in one episode, calculated by summing the queue lengths of all lanes and dividing by the total number of lanes, measured in vehicles.

To evaluate the effectiveness of the PN_D3QN algorithm, it is compared with several representative TSC algorithms.

- FTC: Adopts a predefined timing scheme, with a signal cycle of 80 s.
- Max-Pressure (MP): Selects the phase control signal light with the maximum pressure^18^.
- Dueling Double Deep Q-Network (D3QN): An improved version based on the DQN algorithm^28^.

### Evaluation and analysis of results

**Figure 4:**
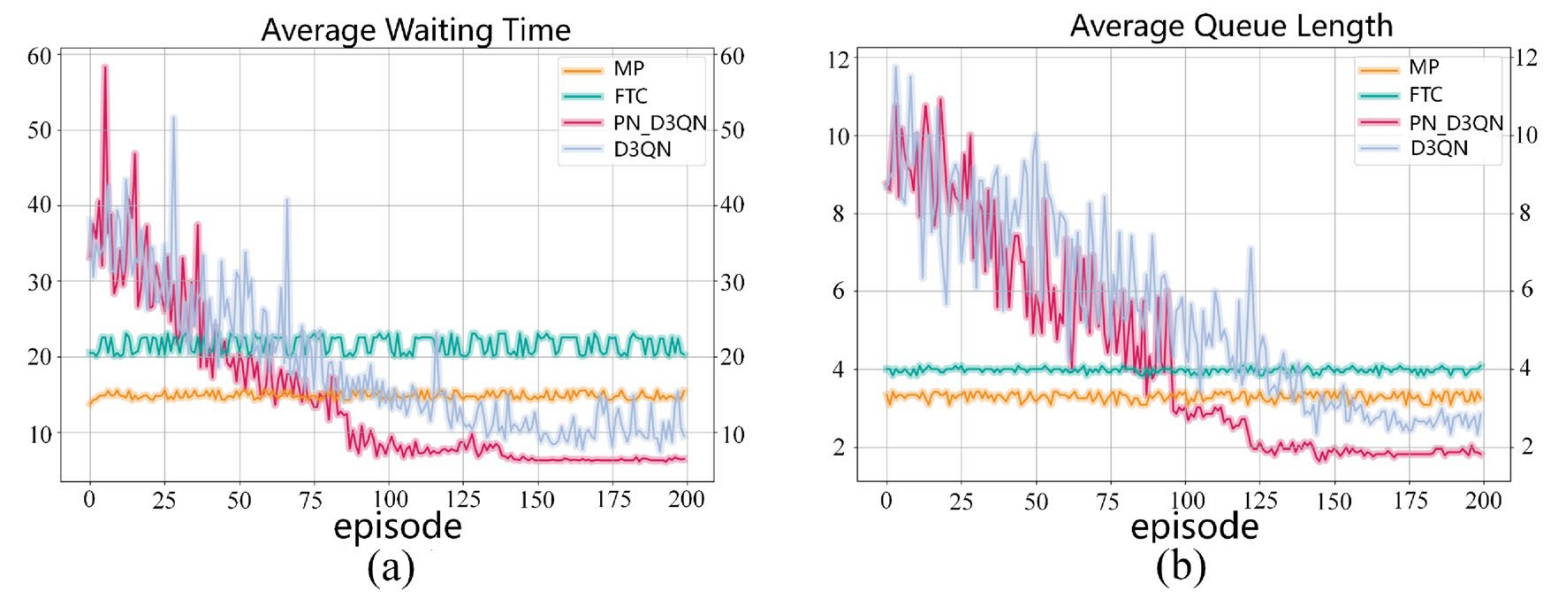
Performance comparison of various algorithms in training result. (**a**) Average waiting time for vehicles. (**b**) Average queue length for lanes.

The model training experiment used traffic flow from Scenario 1. The experimental results are shown in Fig. 4, where the performance of four different traffic signal control algorithms over 200 episodes can be observed. Figure 4a illustrates the average waiting time for vehicles, while Fig. 4b shows the average queue length per lane. Since the MP method is static and lacks learning and adaptation capabilities, its fluctuations are mainly caused by random variations in real-time traffic conditions. On the other hand, FTC is entirely predefined and does not consider changes in traffic flow. Therefore, it exhibits stable but non-adaptive behavior across all episodes without any improvement trend. In contrast, DRL-based D3QN and PN_D3QN methods can continuously interact with the environment to learn and adjust their strategies, which is reflected in the fluctuation of the curves. The initial performance fluctuations are a direct result of the model's trial-and-error exploration for optimal strategies. As training progresses, both methods demonstrate improved learning efficiency, leading to gradually stabilized performance. Especially PN_D3QN, its introduced algorithmic optimizations make the learning process faster, convergence speed quicker, and ultimately achieve more efficient learning of the optimal policy *π*. Upon reaching a stable training state, PN_D3QN exhibits excellent performance in both average waiting time for vehicles and average queue length per lane, surpassing D3QN. Meanwhile, MP and FTC lag behind, with FTC showing the least desirable performance.

**Figure 5:**
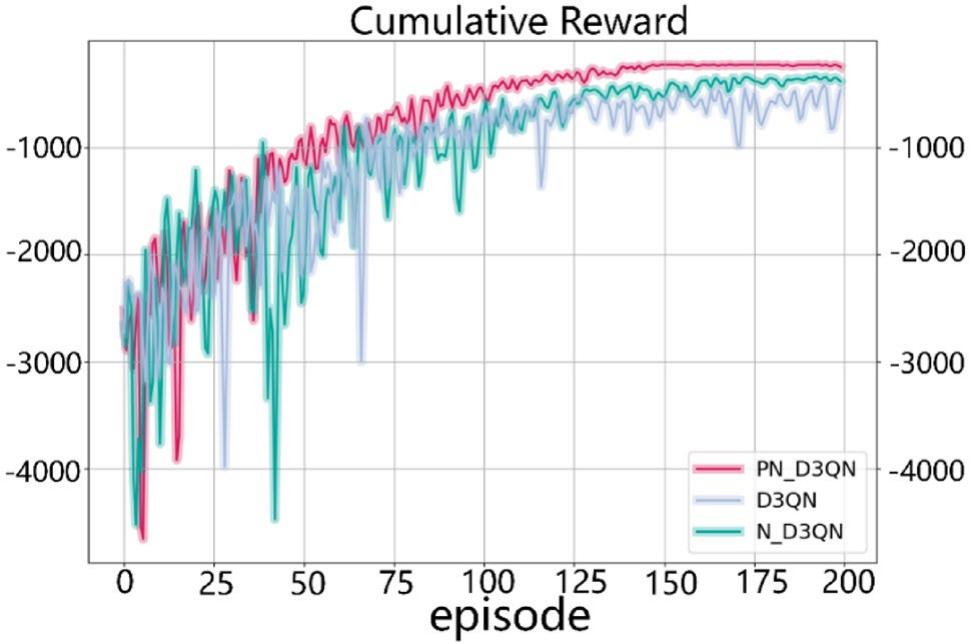
Comparison of cumulative reward.

The model training ablation experiment used traffic flow from Scenario 1, and the experimental results are shown in Fig. 5. Different colored curves represent the performance of different algorithm variants over 200 training episodes, with cumulative reward as the metric. Higher cumulative reward indicates that the algorithm made better decisions in a specific task. The cumulative reward of the PN_D3QN algorithm rapidly increases in the early stages of training and stabilizes later in training. This indicates that PN_D3QN can learn quickly and find effective strategies, maintaining high stability after finding effective strategies. However, the convergence speed of the N_D3QN algorithm is relatively slow due to the lack of priority experience replay mechanism. After removing the noise network, the curve fluctuation of D3QN after convergence is relatively large, indicating poorer stability of its strategy compared to PN_D3QN. Additionally, because there was basically no upward trend in the three curves after training for 175 episodes, the average cumulative rewards of the last 25 episodes were taken. The average cumulative reward values of PN_D3QN, N_D3QN, and D3QN algorithms were − 229.24, − 367.33, and − 592.05, respectively. The reward value of PN_D3QN was significantly improved compared to D3QN. In conclusion, since the PN_D3QN method integrates noise networks and PER techniques on the basis of D3QN, it demonstrates the fastest learning speed and highest stability. This suggests that combining noise networks, PER techniques, dueling network, and double q-learning is effective in TSC tasks.

**Figure 6:**
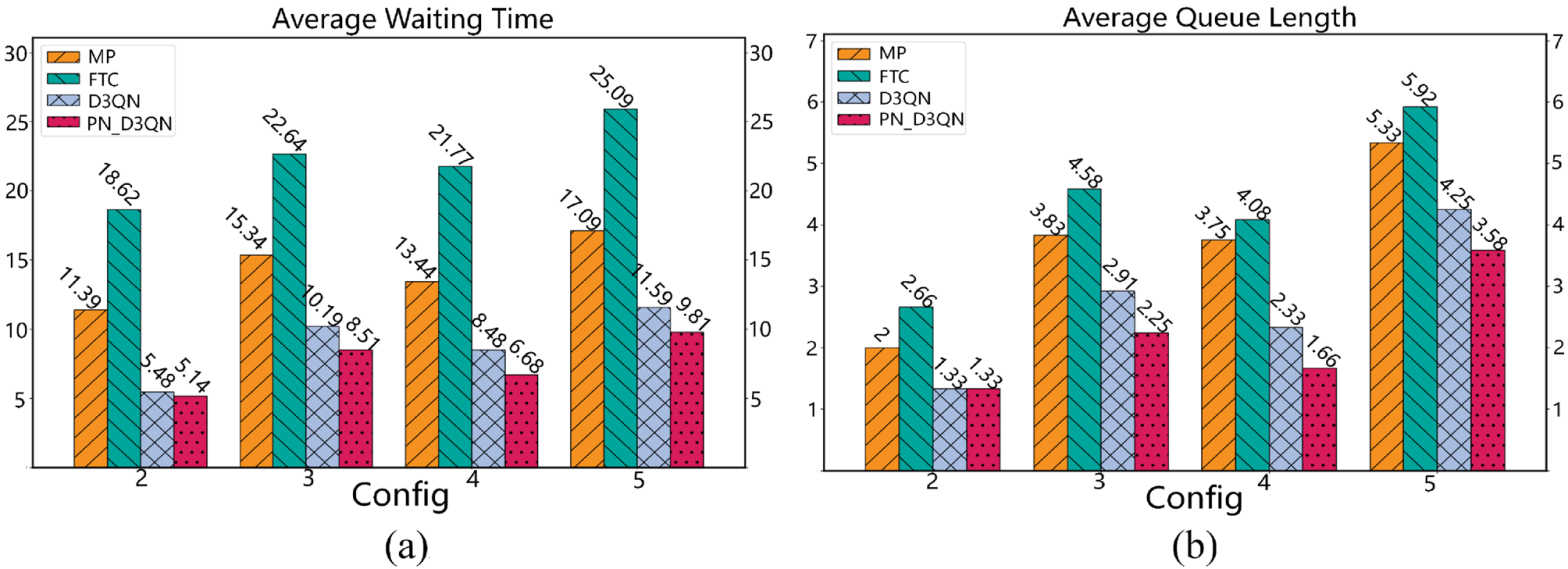
Performance comparison of various algorithms in testing result. (**a**) Average waiting time for vehicles. (**b**) Average queue length for lanes.

To validate the robustness of the trained PN_D3QN model, tests were conducted using traffic flows from scenarios 2, 3, 4, and 5 in Table 2. Both D3QN and PN_D3QN models selected the models saved after 200 episodes for testing, with only one test episode conducted. Data were obtained from lane detectors and averaged cumulatively. Specifically, in scenarios 2 and 3, the arrival rates of traffic from the north–south and east–west directions were consistent, but scenario 2 exhibited lower traffic density compared to scenario 3. The experimental results as shown in Fig. 6, indicated that in these two scenarios, the average waiting time and queue length of the FTC method were significantly higher than other methods, with the MP method following closely. It is worth noting that in the low-traffic-density scenario 2, the performance of D3QN was similar to the optimized PN_D3QN algorithm. However, in the high-density scenario 3, the PN_D3QN algorithm demonstrated significant advantages over the D3QN algorithm in both average waiting time and queue length. Scenario 4 simulated asymmetric high-density traffic flows, where the PN_D3QN algorithm outperformed other methods in reducing average waiting time, with D3QN, MP, and FTC methods following in sequence. The performance of queue length was similar to that of waiting time. Scenario 5 represented a situation with highly complex traffic flow variations. Compared to the MP and FTC methods, the PN_D3QN and D3QN methods exhibited higher traffic efficiency, reflected in shorter average waiting times and queue lengths. The average waiting time of PN_D3QN decreased by 15.35%, 42.59%, and 60.9% compared to D3QN, MP, and FTC, respectively, while the average queue length decreased by 15.76%, 32.83%, and 39.52%. The experimental results highlight the superiority of DRL algorithms in dealing with dynamic and variable traffic flow scenarios, especially the potential of the optimized PN_D3QN algorithm in improving traffic flow efficiency. Therefore, the PN_D3QN method can adapt to different traffic flow scenarios and exhibits better robustness.

## Conclusion

This study proposes an innovative TSC method that integrates dueling network, noise network, PER, and double q-learning mechanisms. This method effectively extracts key features of intersection traffic flows by analyzing discretized state space and utilizes vehicle queue length, which is easier to obtain in practical applications, as the reward function to guide the model training process. Action selection relies on a cyclic phase action space to provide decision support for phase transitions. In this method, PER accelerates the convergence speed of the model through non-uniform sampling; meanwhile, the introduction of noise network enhances the robustness of the model; the application of dueling network and double q-learning effectively alleviates the problem of overestimation that may occur in the DQN algorithm. Through training and ablation experiments in scenario 1, it is verified that this method can quickly learn better decision strategies *π* and exhibit accelerated convergence characteristics. In the testing experiments of scenarios 2–5, it is further demonstrated that this method can signirove traffic flow efficiency and reduce vehicle waiting time and lane queue length in different scenarios.

However, our study still has some limitations and should be further improved in future research. One major drawback of the DRL-based TSC framework is the cold-start problem during early training, where random exploration leads to poor performance, worse than even basic FTC methods. This poses significant challenges for real-world applications. Future solutions may involve using offline DRL or safe DRL. In future research, more field data should be introduced to further validate the model proposed in our study. Additionally, future studies should extend single-agent intersection signal control to multi-agent intersection collaborative signal control.

## Data Availability

The datasets used, generated and analyzed during this study are available from the corresponding author on reasonable request.

## References

[1] Lu J, Li B, Li H (2021). Expansion of city scale, traffic modes, traffic congestion, and air pollution. Cities.

[2] Wei H, Zheng G, Gayah V (2021). Recent advances in reinforcement learning for traffic signal control: A survey of models and evaluation. ACM SIGKDD Explor. Newsl..

[3] Marshall WE, Dumbaugh E (2020). Revisiting the relationship between traffic congestion and the economy: A longitudinal examination of US metropolitan areas. Transportation.

[4] Afrin T, Yodo N (2020). A survey of road traffic congestion measures towards a sustainable and resilient transportation system. Sustainability.

[5] De Oliveira LFP, Manera LT, Da Luz PDG (2020). Development of a smart traffic light control system with real-time monitoring. IEEE Internet Things J..

[6] Wang F, Lai G (2020). Fixed-time control design for nonlinear uncertain systems via adaptive method. Syst. Control Lett..

[7] Eom M, Kim BI (2020). The traffic signal control problem for intersections: A review. Eur. Transport Res. Rev..

[8] Wang T, Cao J, Hussain A (2021). Adaptive traffic signal control for large-scale scenario with cooperative group-based multi-agent reinforcement learning. Transport Res. Part C: Emerg. Technol..

[9] Robertson DI, Bretherton RD (1991). Optimizing networks of traffic signals in real time-the SCOOT method. IEEE Trans. Veh. Technol..

[10] Diakaki C, Papageorgiou M, Aboudolas K (2002). A multivariable regulator approach to traffic-responsive network- wide signal control. Control Eng. Pract..

[11] Oh J, Hessel M, Czarnecki WM (2020). Discovering reinforcement learning algorithms. Adv. Neural Inf. Process. Syst..

[12] Janiesch C, Zschech P, Heinrich K (2021). Machine learning and deep learning. Electron. Markets.

[13] Ladosz P, Weng L, Kim M (2022). Exploration in deep reinforcement learning: A survey. Inf. Fusion.

[14] Gregurić M, Vujić M, Alexopoulos C (2020). Application of deep reinforcement learning in traffic signal control: An overview and impact of open traffic data. Appl. Sci..

[15] Noaeen M, Naik A, Goodman L (2022). Reinforcement learning in urban network traffic signal control: A systematic literature review. Expert Syst. Appl..

[16] Wu C, Kim I, Ma Z (2023). Deep reinforcement learning based traffic signal control: A comparative analysis. Procedia Comput. Sci..

[17] Singh L, Tripathi S, Arora H (2009). Time optimization for traffic signal control using genetic algorithm. Int. J. Recent Trends Eng..

[18] Levin MW, Hu J, Odell M (2020). Max-pressure signal control with cyclical phase structure. Transport. Res. Part C: Emerg. Technol..

[19] Pandit K, Ghosal D, Zhang HM, Chuah CN (2013). Adaptive traffic signal control with vehicular ad hoc networks. IEEE Trans. Veh. Technol..

[20] Kulkarni, G. H. & Waingankar, P. G. Fuzzy logic based traffic light controller. In *2007 International Conference on Industrial and Information Systems. IEEE* 107–110 (2007).

[21] Abdoos, M., Mozayani, N. & Bazzan, A. L. Traffic light control in non-stationary environments based on multi agent Q-learning. In *2011 14th International IEEE Conference on Intelligent Transportation Systems, ITSC, IEEE* 1580–1585 (2011).

[22] El-Tantawy S, Abdulhai B, Abdelgawad H (2014). Design of reinforcement learning parameters for seamless application of adaptive traffic signal control. J. Intell. Transp. Syst. Technol. Plann. Oper..

[23] Medina, J. C. & Benekohal, R. F. Traffic signal control using reinforcement learning and the max-plus algorithm as a coordinating strategy. In *2012 15th International IEEE Conference on Intelligent Transportation Systems, vol. 59, IEEE* 6–601 (2012).

[24] Wiering, M. A. Multi-agent reinforcement learning for traffic light control. In *Machine Learning: Proceedings of the Seventeenth International Conference, ICML’2000* 1151–1158 (2000).

[25] Mnih V, Kavukcuoglu K, Silver D (2015). Human-level control through deep reinforcement learning. Nature.

[26] Wei, H., Chen, C., Zheng, G., Wu, K. & Li, Z. PressLight: Learning max pressure control to coordinate traffic signals in arterial network. In *The 25th ACM SIGKDD International Conference* (ACM, 2019).

[27] Zhang, L., Xie, S & Deng, J. Leveraging queue length and attention mechanisms for enhanced traffic signal control optimization. In *Joint European Conference on Machine Learning and Knowledge Discovery in Databases* 141–156 (Springer, 2023).

[28] Liang X, Du X, Wang G, Han Z (2019). A deep reinforcement learning network for traffic light cycle control. IEEE Trans. Veh. Technol..

[29] Zheng, G. *et al.* Learning phase competition for traffic signal control. In *Proceedings of the 28th ACM International Conference on Information and Knowledge Management, CIKM ’19, ACM* 1963–1972 (2019).

[30] Li SZ, Yu H, Zhang G, Dong S, Xu CZ (2021). Network-wide traffic signal control optimization using a multi-agent deep reinforcement learning. Transp. Res. C.

[31] Ma D, Zhou B, Song X, Dai H (2021). A deep reinforcement learning approach to traffic signal control with temporal traffic pattern mining. IEEE Trans. Intell. Transp. Syst..

[32] Wei, H., Yao, H., Zheng, G. & Li, Z. IntelliLight: A reinforcement learning approach for intelligent traffic light control. In *Proceedings of the 24th ACM SIGKDD International Conference on Knowledge Discovery and Data Mining, KDD 2018* 2496–2505 (2018).

[33] Li D, Wu J, Xu M (2020). Adaptive traffic signal control model on intersections based on deep reinforcement learning. J. Adv. Transport..

[34] Genders, W. & Razavi, S. Using a deep reinforcement learning agent for traffic signal control. arXiv:1611.01142 (2016).

[35] Bouktif S, Cheniki A, Ouni A (2023). Deep reinforcement learning for traffic signal control with consistent state and reward design approach. Knowl. Based Syst..

[36] Kang L, Huang H, Lu W (2023). A dueling deep Q-network method for low-carbon traffic signal control. Appl. Soft Comput..

[37] Wang, Z., Schaul, T., Hessel, M. *et al*. Dueling network architectures for deep reinforcement learning. In *International Conference on Machine Learning. PMLR* 1995–2003 (2016).

[38] Van Hasselt, H., Guez, A. & Silver, D. Deep reinforcement learning with double q-learning. In *Proceedings of the AAAI Conference on Artificial Intelligence, vol. 30* (2016).

[39] Schaul, T., Quan, J., Antonoglou, I. & Silver, D. Prioritized experience replay. In *Proceedings of the international conference on learning representations (ICLR)* (2016).

[40] Han S, Zhou W, Lu J (2022). NROWAN-DQN: A stable noisy network with noise reduction and online weight adjustment for exploration. Expert Syst. Appl..

[41] Puterman ML (1990). Markov decision processes. Handb. Oper. Res. Manage. Sci..

[42] Wu T, Zhou P, Liu K (2020). Multi-agent deep reinforcement learning for urban traffic light control in vehicular networks. IEEE Trans. Veh. Technol..

